# Diagnosis, treatment, and functional outcomes for two adolescent female patients with lupus myelitis: a case report

**DOI:** 10.3389/fresc.2025.1454381

**Published:** 2025-03-14

**Authors:** Deanna Claus, Andrew McCoy, Denesh Ratnasingam, Cristina Saez, Gabriel Tarshish, Cristina Sarmiento

**Affiliations:** ^1^ Department of Physical Medicine and Rehabilitation, University of Colorado Anschutz Medical Campus, Aurora, CO, United States; ^2^ Division of Rehabilitation Medicine, Children's Hospital of Philadelphia, Philadelphia, PA, United States; ^3^ Division of Pediatric Rehabilitation Medicine, Children's Hospital Colorado, Aurora, CO, United States; ^4^ Department of Pediatrics, Division of Rheumatology, University of Colorado Anschutz Medical Campus, Aurora, CO, United States

**Keywords:** transverse myelitis, systemic lupus erythematosus, lupus myelitis, spinal cord injury, rheumatology, pediatric rehabilitation, case report

## Abstract

**Introduction:**

Transverse myelitis is a rare neurologic complication associated with systemic lupus erythematosus (SLE), also known as lupus myelitis. Little is known about the optimal treatment regimen for the disease or the functional outcomes after diagnosis, especially for pediatric patients.

**Methods:**

A retrospective case series at a large, academic pediatric tertiary care center was performed to describe the clinical presentation, diagnostic approach, early treatment, and functional outcomes in two pediatric patients diagnosed with lupus myelitis as a presenting sign of new-onset SLE.

**Results:**

Description of baseline patient characteristics, presenting symptoms and clinical features, laboratory work-up and neuroimaging findings, immunomodulatory therapy, complications, and rehabilitation functional outcomes are described for two adolescent patients diagnosed with lupus myelitis. Both patients presented with features that were initially mistaken for other neurologic conditions. The combination of longitudinally extensive lesions of the spinal cord on neuroimaging and laboratory findings suggestive of an autoimmune process ultimately led to the diagnoses of lupus myelitis and new-onset SLE. Both patients received intravenous and oral corticosteroids, plasmapheresis, rituximab, cyclophosphamide, intravenous immunoglobulin, and acute intensive rehabilitation including physical therapy, occupational therapy, and speech therapy. Both patients demonstrated marked functional improvement in domains of self-care and mobility in the setting of acute inpatient rehabilitation.

**Discussion:**

While this diagnosis has been described in adult literature, there is limited evidence regarding management or functional outcomes for pediatric cases of lupus myelitis. Collaboration between rheumatology and rehabilitation teams allowed for a coordinated approach to achieve medical and functional goals. Early diagnosis, treatment, and acute inpatient rehabilitation led to significant improvement in functional outcomes for the two pediatric patients in this study.

## Introduction

Neuropsychiatric involvement has been reported in more than half of patients with SLE (1, 2) and is present in 13%–45% of children with SLE (3). It can manifest with a broad range of symptoms from headache or peripheral neuropathy to stroke or seizure (1, 3, 4). Acute transverse myelitis (ATM) due to systemic lupus erythematosus (SLE), also known as lupus myelitis, is a rare neurologic complication that is estimated to occur in 0.7% of patients with SLE, only 1%–2% of whom are pediatric (5, 6). Lupus myelitis even less commonly presents as an initial manifestation of SLE and often presents years after initial diagnosis (4, 5). Those affected are predominantly female (77%) with an average age of 36 years, but it has been reported in patients as young as 5 years old (3, 5). Lupus myelitis most commonly presents as longitudinally extensive transverse myelitis (LETM) (1), with evidence of damage to the spinal cord that extends 3 or more vertebral levels on magnetic resonance imaging (MRI). Overall, relatively little is known about this rare complication of SLE, and even less has been described in the pediatric population.

While there is still no standard treatment protocol, proposed treatment options are largely based on the treatment approach to generalized SLE. See Supplementary Table 1 for immunosuppression protocols. Suggestions for treatment have included, but are not limited to, high-dose methylprednisolone, intravenous immunoglobulin (IVIG), plasmapheresis, cyclophosphamide, and rituximab (3, 6, 7–9). Additionally, little is known about the functional recoveries of these patients, and it is unclear if functional outcomes from lupus myelitis can be compared to transverse myelitis from other causes. Even less is documented about the recovery of pediatric patients with this condition. Data primarily focused on adults have previously shown that only 16% of patients recover to a premorbid state (5) and up to half of patients with lupus myelitis experience a recurrence (1). However, it is hypothesized that the epidemiologic, clinical, and radiographic data for pediatric cases of transverse myelitis (of any cause) are distinct from the adult population, and therefore it is challenging to apply findings from the adult population to children (4). This paucity of data leaves questions about the overall expected trajectory for pediatric patients diagnosed with this condition and how to appropriately counsel patients and families. The objective of this case series was to describe both the acute phase of management, from diagnosis to treatment, and the detailed functional outcomes at time of admission and discharge from inpatient rehabilitation for two pediatric patients with lupus myelitis as their initial presentation of SLE.

## Case descriptions

**Figure 1:**
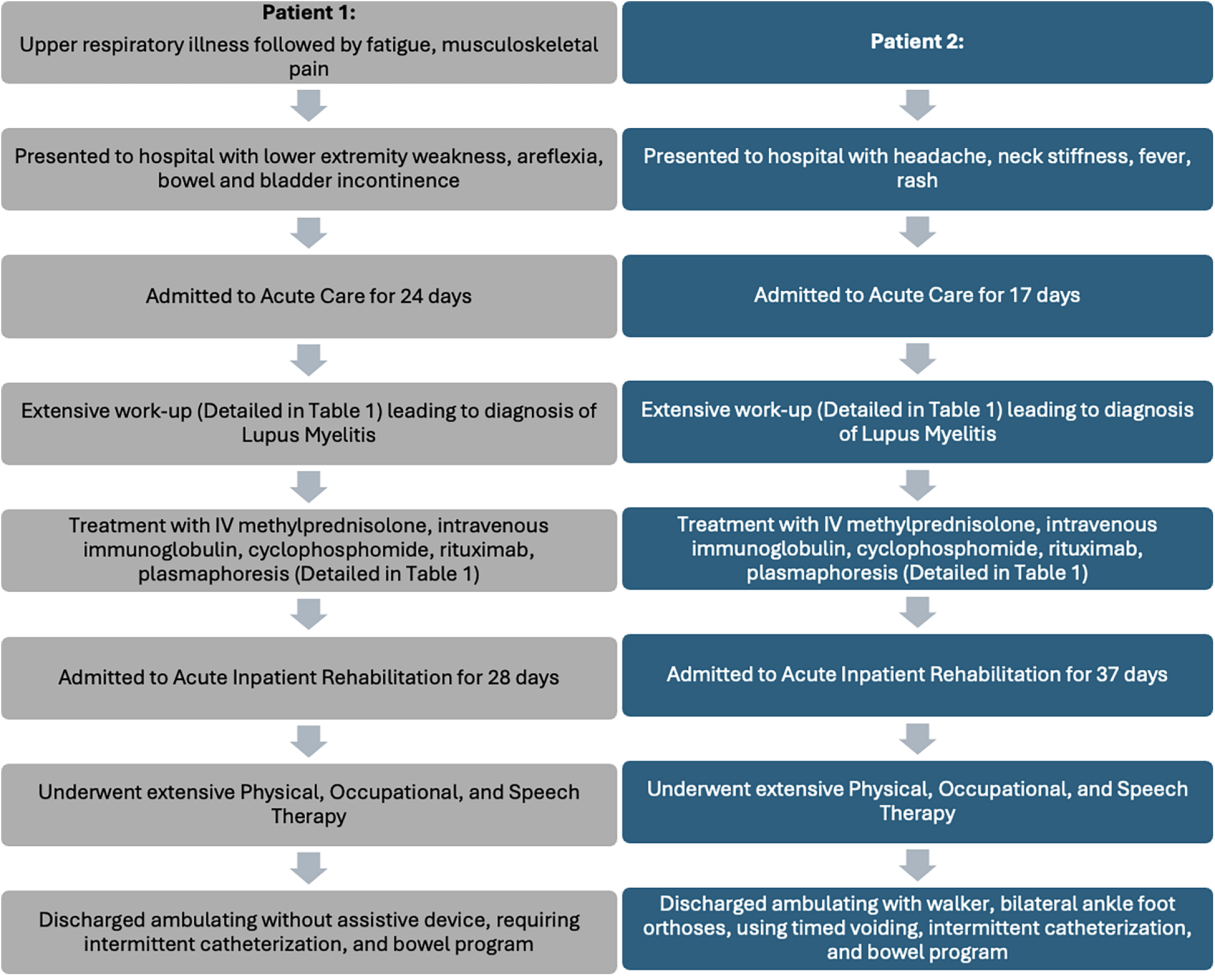
Case series timeline: timeline of presentation, acute care management, and acute inpatient rehabilitation for adolescent patients (*N* = 2) with lupus myelitis.

**Figure 2:**
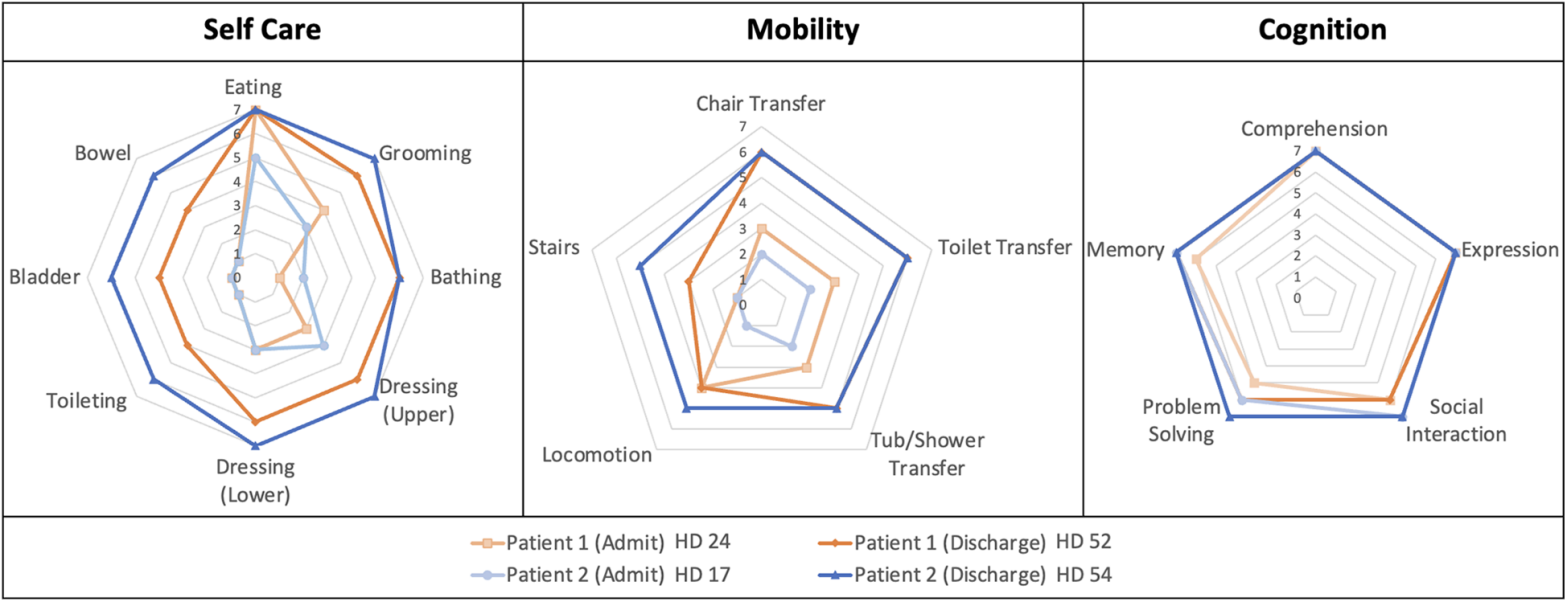
Functional independence measure for children (WeeFIM) outcomes on admission and discharge from inpatient rehabilitation. The WeeFIM functional outcomes are represented as the three subdomains of the scoring system with Patient 1 (orange) and Patient 2 (blue) demonstrating substantial functional gains within the Self-Care and Mobility domains.

This retrospective case series describes two patients with lupus myelitis who presented to our institution between March 2021 and December 2022. The Institutional Review Board at the University of Colorado deemed our case series to be exempt from review, though we obtained both parental written consent and patient assent for inclusion in this case series. Both patients underwent extensive diagnostic evaluation and treatment, then completed acute inpatient rehabilitation, led by a pediatric rehabilitation medicine team. Inpatient rehabilitation consisted of intensive physical and occupational therapy, as well as speech therapy and rehabilitation neuropsychology for supported learning and coping during their recovery. Additionally, they received support from rehabilitation nursing, case management, and social work. Figure 1 describes a general timeline and details on both the acute and rehabilitation phases of care. Patient demographics and clinical characteristics are summarized in Table 1. Table 1 also includes details on rheumatologic treatments used. Response to treatment was tracked with regular laboratory monitoring and functional recovery was followed with the Functional Independence Measure for Children (WeeFIM). The WeeFIM is a pediatric version of the adult Functional Independence Measure and validated to describe functional outcomes in pediatric patients undergoing rehabilitation (10). The WeeFIM scores describe the level of assistance, from 1 (totally dependent) to 7 (totally independent), required to complete tasks in three subdomains including self-care, mobility, and cognition. We have represented WeeFIM functional outcomes for each patient on admission to and discharge from inpatient rehabilitation in Figure 2. Definitions of WeeFIM scores and raw data from each patient's WeeFIM are available in Supplementary Tables 2 and 3.

**Table 1:** Timeline and characteristics of patients (*N* = 2), acute care management, and acute inpatient rehabilitation.

Patient characteristic or variable	Patient 1	Patient 2
Demographic characteristics		
Age (years)	12	15
Gender (assigned at birth)	Female	Female
Past medical history	None	Aseptic meningitis at age 9, Depression
Prodromal signs and symptoms	Preceding upper respiratory illness, fatigue, musculoskeletal pain	Headache, neck stiffness, fever, rash
Acute care management		
Length of acute care admission	24 days	17 days
Clinical findings on presentation	Urinary retention, lower extremity weakness, areflexia	Lower extremity weakness, areflexia, bowel and bladder incontinence
MRI findings	T2 hyperintensity nearly the entire length of spinal cord, greatest in central gray matter, including non-enhancing T2 hyperintensity in the upper cervical spinal cord extending into the cervicomedullary junction	Scattered T2 signal abnormalities throughout, extending from T1 to conus medullaris; faint cranial nerve (V, VI, VII) and increased sulcal enhancement in brain
Cerebrospinal fluid findings	WBC count: 93 cells/mm^3^ Glucose: 22 mg/dl Protein: >200 mg/dl	WBC count: 119 cells/mm^3^ Glucose: 39 mg dl Protein: 49 mg dl
SLICC 2012 Classification Criteria for SLE	Clinical Criteria: Neurologic disease Lymphopenia (ALC 930), Thrombocytopenia (80,000) Immunologic Criteria:+ANA+Anti-dsDNA Ab+Anti-Smith Ab+Beta 2 glycoprotein Ab Hypocomplementemia	Clinical Criteria: Neurologic disease Immunologic Criteria:+ANA+Anti-dsDNA Ab + Coombs Hypocomplementemia
Other pertinent diagnostic work up	− Serum NMO IgG Elevated ESR and CRP+ENA Ab+Antiphospholipid Ab−RF	− Serum NMO IgG Elevated ESR and CRP+ENA Ab−Antiphospholipid Ab−RF
Acute immunomodulatory treatment	IV methylprednisolone 1,000 mg (HD # 8, 9, 10) IVIG 0.765 g/kg (HD # 4, 5, 6) Cyclophosphamide 500 mg (HD # 10, 23, 37, 50, 71) Rituximab induction 1,000 mg (HD # 9, 24) Plasmapheresis (HD # 13, 15, 17, 22)	Corticosteroids IV 1,000 mg (HD # 3, 4, 5, 6, 7) IVIG 1 g/kg (HD # 64,65) Cyclophosphamide 1,200 mg (750/m^2^) (HD # 12, 41, 66, 82, 123, 151) Rituximab induction 1,000 mg (HD # 14, 27) Plasmapheresis (HD # 4, 5, 7, 9, 11)
Non-CNS complications during acute care admission	Vitamin D deficiency associated hypophosphatemia Hyponatremia Pseudomonal UTI	Vitamin D deficiency associated hypophosphatemia Hypertension
Post-Acute Rehabilitation		
Length of rehabilitation admission	28 days	37 days
Neurologic level of injury	C3 incomplete tetraplegia	T10 incomplete paraplegia
Nutrition	Oral diet, regular solids, thin liquids	Oral diet, regular solids, thin liquids
Bowel management	Daily suppository without digital stimulation	Daily suppository with digital stimulation
Bladder management	Timed voids with additional intermittent catheterization	Intermittent catheterization, oxybutynin
Non-CNS complications during rehabilitation admission	Klebsiella UTI	Non-occlusive DVT
Discharge planning		
Orthotics	Bilateral AFOs	None
Medical supplies	Catheter and bowel care supplies	Catheter and bowel care supplies
Durable medical equipment	Front wheeled walker, shower chair	Rental manual wheelchair, shower chair
Outpatient therapies	PT, OT, and pelvic floor therapy	PT, pelvic floor therapy
School Accomodations/Services	Yes	Yes

AFO, ankle foot orthoses; Ab, antibody; Anti-dsDNA, anti-double stranded deoxyribonucleic acid; ANA, antinuclear antibody; Anti-RNP, antinuclear ribonucleoprotein; CRP, C reactive protein; CSF, cerebral spinal fluid; DVT, deep vein thrombosis; ENA, extractible nuclear antigen; ESR, erythrocyte sedimentation rate; IVIG, intravenous immunoglobulin; MRI, magnetic resonance imaging; NMO IgG, neuromyelitis optica immunoglobulin G; OT, occupational therapy; PT, physical therapy; RF, rheumatoid factor; SSA, Sjögren's-syndrome related antibody A; UTI, urinary tract infection; WBC, white blood cell.

### Case 1

Patient 1 was a 12-year-old female with no significant past medical history who presented with one week of fatigue, neck, back, and lower extremity pain, as well as urinary retention in the setting of an upper respiratory infection three weeks prior. Initial work-up was notable for urinalysis suggestive of urinary tract infection (UTI) with signs of pyelonephritis and mild bilateral hydronephrosis on renal ultrasound. She was started on antibiotic treatment and admitted to the hospital. The following day, she developed bilateral lower extremity and right arm weakness and was noted to have difficulty transferring from bed. Her progressive weakness was associated with areflexia in the lower extremities and hyperreflexia in the upper extremities. She was initially suspected to have acute flaccid myelitis.

**Figure 3:**
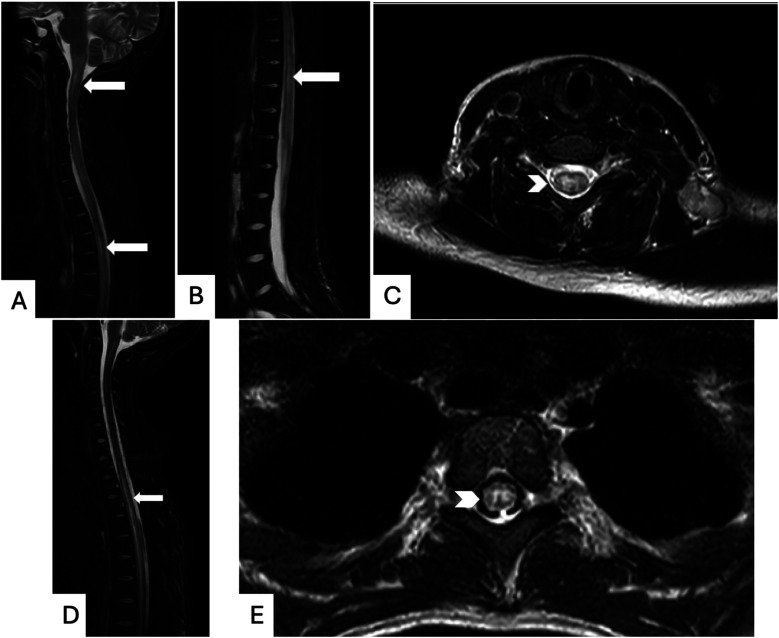
Magnetic resonance imaging (MRI) of the spine: patient 1: T2-weighted MRI images without contrast demonstrating longitudinally extensive T2 hyperintensities in the cervicomedullary junction through cervical and thoracic **(A)** and lumbar **(B)** spinal segments (long white arrows), as well as hyperintense signal on axial MRI cervical spine **(C)** affecting both gray and white matter (thick white arrowhead). Patient 2: T2-weighted Fluid-attenuated inversion recovery (FLAIR) sequence MRI images with Gadolinium contrast demonstrating extensive T2 hyperintensities in the thoracic spinal cord both on sagittal **(D)** and axial **(E)** views, demonstrated with long white arrows and thick white arrowhead, respectively.

The care team obtained MRI of the brain and spinal cord, which showed a non-enhancing T2 hyperintensity in the upper cervical spinal cord extending into the cervicomedullary junction as well as a long segment of T2 hyperintensities extending nearly the entire length of her spinal cord, greatest within the central gray matter (Figures 3A–C). Lumbar puncture was performed, and cerebrospinal fluid (CSF) analysis revealed low glucose, elevated protein, and pleocytosis (Table 1). Initial treatment with intravenous immunoglobulin (IVIG) for three days for presumed acute flaccid myelitis resulted in improvement in urinary retention and extremity weakness.

Autoimmune work-up was initiated due to elevated inflammatory markers and pancytopenia and was notable for positive antinuclear antibody (ANA), anti-double stranded deoxyribonucleic acid (anti-dsDNA), anti-Smith, and antinuclear ribonucleoprotein (RNP) antibodies, elevated erythrocyte sedimentation rate (ESR), and hypocomplementemia. These laboratory and imaging findings met Systemic Lupus International Collaborating Clinics (SLICC) classification criteria for a new diagnosis of SLE complicated by lupus myelitis (see Table 1) (11). Diagnosis was formally made on day 10 of the inpatient stay.

Treatment then shifted to 1,000 mg methylprednisolone IV for 3 doses, plasmapheresis, rituximab, and cyclophosphamide, which led to normalization of complements, cell counts, and ESR, in addition to clinical improvement in lower extremity weakness. Her lower extremity areflexia transitioned to hyperreflexia approximately 4 days after initiation of treatment, consistent with the typical evolution in transverse myelitis.

Pediatric rehabilitation medicine evaluation demonstrated a C3 neurologic level of injury with tetraplegia. After 19 days in acute care, she was admitted to inpatient rehabilitation for 28 days while continuing treatment for SLE. She demonstrated substantial improvement in total WeeFIM score from 66 to 100 (out of 126); see Figure 2. She had the most substantial improvements in the self-care subdomain, improving from 21 to 43 (out of 56), and improving from 14 to 24 (out of 35) in the mobility subdomain. Specifically, regarding self-care, she improved from a total assistance level to a minimum assistance level for bathing, toileting, and bowel and bladder management. Her bladder management program consisted of scheduled clean intermittent catheterization three times daily and bowel management required oral medications and a scheduled evening suppository. Related to mobility, she improved from a moderate level of assistance for transfers and bed-level mobility to modified independence with transfers and modified independence with ambulation using bilateral ankle-foot orthotics (AFOs) and a forward wheeled walker. At discharge, she had normal upper extremity strength and distal more than proximal lower extremity paresis.

As an outpatient, she continued to follow with rheumatology and completed the Euro-Lupus cyclophosphamide protocol (500 mg every two weeks for 12 weeks) (12) followed by a transition to mycophenolate mofetil. She was maintained on hydroxychloroquine and prednisone as well. She was seen in the rehabilitation medicine clinic after three weeks where she ambulated using AFOs and a walker.

### Case 2

Patient 2 was a 15-year-old female with history of depression who presented to the hospital with fever, headache, neck stiffness, and rash with concern for aseptic meningitis, based on CSF pleocytosis and negative infectious evaluation. Approximately four days after initial symptoms, she developed acute lower extremity paralysis, areflexia, and loss of bowel and bladder function.

Neurologic consultation recommended MRI of the brain and entire spine with and without contrast, which demonstrated scattered T2 signal abnormalities throughout the spinal cord extending from T1 to the conus medullaris (Figure 3 D and E). She received five days of plasmapheresis and five days of 1,000 mg methylprednisolone IV with subtle improvement in her symptoms. Given elevated inflammatory markers concerning for possible autoimmune process, rheumatologic evaluation was initiated and significant for positive ANA, dsDNA, anti-Sjögren's Syndrome Related Antigen A, anti-ribonucleoprotein (RNP), and anti-Smith autoantibodies in addition to hypocomplementemia. Laboratory findings in conjunction with neuroimaging findings met SLICC classification criteria for a diagnosis of SLE complicated by lupus myelitis (see Table 1). Diagnosis was formally made on day 11 of her admission. She received cyclophosphamide and rituximab after diagnosis of SLE was established and continued with weekly doses of 1,000 mg methylprednisolone IV.

Pediatric rehabilitation medicine examination demonstrated a T10 neurologic level of injury with paraplegia. After 17 days in acute care, she was admitted to inpatient rehabilitation for 37 days and continued treatment for SLE. The rehabilitation course was complicated by a provoked venous thromboembolism due to a peripherally inserted central catheter, and she was treated with 3 months of enoxaparin. As shown in Figure 2, she demonstrated improvement in her total WeeFIM score from 62 to 114 (out of 126) with most improvements in the self-care subdomain (21–52, out of 56) and mobility subdomain (8–27, out of 35). Regarding self-care, she initially required moderate to maximum assistance with bathing and grooming and minimum to moderate assistance with dressing at the time of rehabilitation admission. By discharge, she was independent with these tasks, except for bathing (modified independent). She improved from requiring total assistance to modified independence for toileting and bowel and bladder management. Her bladder program consisted of clean intermittent catheterization every four hours during the day and every six hours at night with the addition of oxybutynin to treat bladder spasticity. Her bowel program consisted of a scheduled evening suppository with digital stimulation and oral medications. Related to mobility, she improved from requiring maximum assistance to modified independence in all transfer categories, except for tub/shower transfers which required supervision. She additionally improved from requiring total assistance for ambulation to supervision with all ambulation including stairs, without assistive devices or orthotics.

For disease modifying treatment, with the guidance of the rheumatology team, she received the Cytoxan NIH protocol (750 mg/m^2^ every month for six months) and continued on azathioprine and hydroxychloroquine long-term (13). By the time of outpatient rehabilitation medicine clinic follow-up seven weeks after discharge, she was ambulating independently with mild right lower extremity weakness.

## Discussion

Acute transverse myelitis from SLE is thought to be due to either a vascular insult and/or autoimmune phenomenon. Some studies propose that the spinal cord damage is secondary to ischemia, either from vasculitis (1) or from thrombotic vasculopathy secondary to anti-phospholipid antibodies affecting the vasculature of the spinal cord (6). It is also possible that anti-phospholipid antibodies cross react with spinal cord phospholipids and lead to subsequent spinal cord damage (6).

A high index of suspicion is required to identify the condition, especially in pediatric patients. Lupus myelitis has two different phenotypes and can present as a predominantly grey matter myelitis or predominantly white matter myelitis. Correlation between MRI findings and clinical presentation is poor (4, 5). The grey matter myelitis subtype is often characterized by a viral prodrome, lower motor signs including flaccid extremities and hyporeflexia, longitudinally extensive lesions with more rapid deterioration, severe weakness, and greater risk of long-term disability, though recurrence of symptoms is rare (4). The white matter myelitis subtype is characterized less frequently by a viral prodrome and typically is associated with upper motor neuron signs, less severe decline in function over a longer period of time, and less residual weakness. However, this subtype has a greater risk of recurrence, up to 70% (4).

Data reviewing treatment success of pediatric lupus myelitis is limited with mixed results. Most studies cite variable success with intravenous (IV) steroids and cyclophosphamide with or without plasmapheresis (3, 6, 7). Studies seem to agree that early initiation of IMT within 2 weeks of symptom onset is associated with better outcomes, though there is wide variability in treatment regimens (14). Furthermore, outcomes data are also mixed regarding the patient characteristics, laboratory, or imaging findings that may be predictive of a better or worse recovery. One systematic review suggested that patients with higher ESR were more likely to have recovery, while patients with a detectable anti-cardiolipin antibody were more likely to have a poor prognosis (5). In another study, severe neurologic deficits were associated with LETM), lower motor neuron features, and sphincteric dysfunction at the onset of presentation, as well as anti-phospholipid antibody detection (4). Other reports have not found this association and report no prognostic association with age, gender, SLE duration, disease activity, sensory level, association with other CNS involvement, CSF or serology markers (14).

While difficult, it is necessary, to more clearly characterize recovery trajectories after lupus myelitis. In a 2022 systematic review of 102 patients, approximately 5% of patients died, 43% of patients improved, 29% of patients had residual paraplegia, and 17% of patients recovered (5). A different study found that, out of 15 total patients, 3 had complete resolution of symptoms, 6 had good functional improvements, 5 had a good to fair outcome with some functional deficit, and 1 patient died after multiple episodes of recurrent myelitis with development of severe-ventilator dependent tetraplegia (15). However, a significant challenge in interpreting and generalizing these data are the broad, non-specific outcomes used (e.g., “good functional improvement”), which do not clearly define what constitutes these outcomes. Individual case studies and smaller case series paint a more detailed picture of functional recoveries, though they are limited by small sample sizes (14, 16, 17).

Though also limited by a small number of patients and the retrospective data collection performed in this study, these two patients demonstrated significant laboratory and functional improvement with aggressive immunomodulatory treatment (IMT) and intensive inpatient rehabilitation. The inclusion of WeeFIM scores is a strength of this study, as they are an objective measure and can demonstrate trends with time.

This case series describes the presentation, diagnostic evaluation, treatment, and functional outcomes of two adolescent female patients diagnosed with lupus myelitis as a first manifestation of new-onset SLE. There are many similarities between the two presented cases in this study. Both patients were adolescent females without significant prior medical history who presented with urinary retention and lower extremity weakness. Each was initially suspected to have an alternative diagnosis, such as acute flaccid myelitis or aseptic meningitis. Both patients had CSF pleocytosis and longitudinally extensive MRI lesions, though the location of the lesions were different. Of note, though visual evoked potentials can be helpful in the diagnostic work-up, neither patient had these performed. Each patient received plasmapheresis, IV steroids, cyclophosphamide, and rituximab. Of note, patient 2 did not initially experience clinical improvement with IV steroids and plasmapheresis and received rituximab and cyclophosphamide 2 weeks later, whereas patient 1 received most of her IMT within 5 days of diagnosis. It is difficult to say which specific therapies contributed most significantly to clinical improvement. However, both patient outcomes support the use of early aggressive IMT in patients with lupus myelitis. This was tolerated well by both patients. Furthermore, rehabilitation teams focused on improving function and independence while navigating the complications associated with a non-traumatic spinal cord injury. Acute rehabilitation admission also facilitated discharge planning for appropriate bracing, equipment, and therapy needs. Collaboration was required between the rheumatology and rehabilitation teams to coordinate rehabilitation interventions with IMT. Despite having tetraplegia (patient 1) and paraplegia (patient 2), both patients regained strength during their stay and were able to ambulate at the time of discharge either with or without assistive devices. They both required intermittent catheterization and a daily suppository to maintain bladder and bowel continence, respectively. Overall, each patient had significant improvements in their function and independence through coordinated interdisciplinary treatment.

There is limited data regarding pediatric cases of lupus myelitis. Little is known about optimal treatment regimens or expected functional outcomes. Both patients in this case series presented with functional impairments related to new, longitudinally extensive lesions of the spinal cord secondary to lupus myelitis. Their recovery was facilitated by close collaboration between the pediatric rheumatologists and rehabilitation medicine physicians. Early aggressive treatment and a multidisciplinary approach led to improved functional independence for both patients by time of discharge from the hospital.

## Data Availability

The original contributions presented in the study are included in the article/Supplementary Material, further inquiries can be directed to the corresponding author.

## References

[1] Mehmood T Munir I Abduraimova M Antonio Ramirez M Paghdal S McFarlane I M. Longitudinally extensive transverse myelitis associated with systemic lupus erythematosus: a case report and literature review. Am J Med Case Rep. (2019) 7(10):244–9. 10.12691/ajmcr-7-10-6

[2] Unterman A Nolte JES Boaz M Abady M Shoenfeld Y Zandman-Goddard G. Neuropsychiatric syndromes in systemic lupus erythematosus: a meta-analysis. Semin Arthritis Rheum. (2011) 41(1):1–11. 10.1016/j.semarthrit.2010.08.001 20965549

[3] Vieira JP Ortet O Barata D Abranches M Gomes JM. Lupus myelopathy in a child. Pediatr Neurol. (2002) 27(4):303–6. 10.1016/S0887-8994(02)00439-3 12435571

[4] Beh SC Greenberg BM Frohman T Frohman EM. Transverse myelitis. Neurol Clin. (2013) 31(1):79–138. 10.1016/j.ncl.2012.09.008 23186897 PMC7132741

[5] Wen X Xu D Yuan S Zhang J. Transverse myelitis in systemic lupus erythematosus: a case report and systematic literature review. Autoimmun Rev. (2022) 21(6):103103. 10.1016/j.autrev.2022.103103 35452852

[6] Sridhar A Ganesan S Hussain N Shivamurthy V Khan A. Acute longitudinal myelitis as the first presentation in child with systemic lupus erythematosus. J Pediatr Neurosci. (2013) 8(2):150. 10.4103/1817-1745.117854 24082938 PMC3783727

[7] Baca V Sanchez-Vaca G Martínez-Muñiz I Ramírez-Lacayo M Lavalle C. Successful treatment of transverse myelitis in a child with systemic lupus erythematosus. Neuropediatrics. (1996) 27(1):42–4. 10.1055/s-2007-973746 8677025

[8] Dale RC Brilot F Duffy LV Twilt M Waldman AT Narula S Utility and safety of rituximab in pediatric autoimmune and inflammatory CNS disease. Neurology. (2014) 83(2):142–50. 10.1212/WNL.0000000000000570 24920861 PMC4117174

[9] Magro-Checa C Zirkzee EJ Huizinga TW Steup-Beekman GM. Management of neuropsychiatric systemic lupus erythematosus: current approaches and future perspectives. Drugs. (2016) 76(4):459–83. 10.1007/s40265-015-0534-3 26809245 PMC4791452

[10] Ziviani J Ottenbacher KJ Shephard K Foreman S Astbury W Ireland P. Concurrent validity of the Functional Independence Measure for Children (WeeFIM) and the Pediatric Evaluation of Disabilities Inventory in children with developmental disabilities and acquired brain injuries. Phys Occup Ther Pediatr. (2001) 21(2-3):91–101. 10.1300/J006v21n02_08 12029858

[11] Petri M Orbai AM Alarcón GS Gordon C Merrill JT Fortin PR Derivation and validation of the Systemic Lupus International Collaborating Clinics classification criteria for systemic lupus erythematosus. Arthritis Rheum. (2012) 64(8):2677–86. 10.1002/art.34473 22553077 PMC3409311

[12] Houssiau FA Vasconcelos C D’Cruz D Sebastiani GD Garrido ER Danieli MG Immunosuppressive therapy in lupus nephritis: the Euro-Lupus Nephritis Trial, a randomized trial of low-dose versus high-dose intravenous cyclophosphamide. Arthritis Rheum. (2002) 46(8):2121–31. 10.1002/art.10461 12209517

[13] Austin HA Klippel JH Balow JE le Riche NG Steinberg AD Plotz PH Therapy of lupus nephritis. Controlled trial of prednisone and cytotoxic drugs. N Engl J Med. (1986) 314(10):614–9. 10.1056/NEJM198603063141004 3511372

[14] Lu X Gu Y Wang Y Chen S Ye S. Prognostic factors of lupus myelopathy. Lupus. (2008) 17(4):323–8. 10.1177/0961203307088005 18413414

[15] D’Cruz DP Mellor-Pita S Joven B Sanna G Allanson J Taylor J Transverse myelitis as the first manifestation of systemic lupus erythematosus or lupus-like disease: good functional outcome and relevance of antiphospholipid antibodies. J Rheumatol. (2004) 31(2):280–5. 14760797

[16] Jackson SD Wiering BA Bentley IA Herrmann AA Hanson LR. From A to D: a unique case report of recovery after longitudinal myelitis related to lupus. Am J Phys Med Rehabil. (2018) 98(10):e119–22. 10.1097/PHM.0000000000001117 30557157

[17] Campana A Buonuomo PS Insalaco A Bracaglia C Capua MD Cortis E Longitudinal myelitis in systemic lupus erythematosus: a paediatric case. Rheumatol Int. (2012) 32(8):2587–8. 10.1007/s00296-011-2061-1 21792641

