# Supplementary material for: Diagnosis, treatment, and functional outcomes for two adolescent female patients with lupus myelitis: a case report
Source: Front Rehabil Sci. 2025 Mar 14;6:1454381. doi: 10.3389/fresc.2025.1454381 (PMC11949943; doi:10.3389/fresc.2025.1454381)
Supplement: Supplementary file 1 [file Datasheet1.docx]

Supplementary Material

**Supplementary Section and Tables**

**SUPPLEMENTARY TABLE 1:** Lupus Immunosuppression Treatment Protocols

| **NIH Protocol** |
| --- |
| **https://www.ncbi.nlm.nih.gov/pmc/articles/PMC5127968/** |
| **European Protocol** |
| **https://ard.bmj.com/content/78/6/736** |

**SUPPLEMENTARY TABLE 2:** Functional Independence Measure for Children (WeeFIM) Measure of Function

| **Level of Function** | **Assistance Needed** |
| --- | --- |
| 1 | Total assistance (patient performs less than 25% of activity) |
| 2 | Maximal assistance (patient performs between 25% and 49% of activity) |
| 3 | Moderate assistance (patient performs between 50 and 74% of activity) |
| 4 | Minimal assistance (patient performs greater than 75% of activity) |
| 5 | Supervision (patient can perform without assistance, but not independently) |
| 6 | Modified independent (patient performs independently with assistive device) |
| 7 | Independent (patient performs independently in timely and safe manner) |

**SUPPLEMENTARY TABLE 3: Functional Independence Measure for Children (WeeFIM) Outcome Raw Data**

|  | **Patient 1** | | **Patient 2** | |
| --- | --- | --- | --- | --- |
|  | Admission | Discharge | Admission | Discharge |
| **Self-Care** | | | | |
| Eating | 7 | 7 | 5 | 7 |
| Grooming | 4 | 6 | 3 | 7 |
| Bathing | 1 | 6 | 2 | 6 |
| Dressing – Upper Body | 3 | 6 | 4 | 7 |
| Dressing – Lower Body | 3 | 6 | 3 | 7 |
| Toileting | 1 | 4 | 1 | 6 |
| Bladder Management | 1 | 4 | 1 | 6 |
| Bowel Management | 1 | 4 | 1 | 6 |
| **Mobility** | | | | |
| Chair Transfer | 3 | 6 | 2 | 6 |
| Toilet Transfer | 3 | 6 | 2 | 6 |
| Tub / Shower Transfer | 3 | 5 | 2 | 5 |
| Locomotion | 4 | 4 | 1 | 5 |
| Locomotion Description  (Walk, Wheelchair, Crawl, Bed Mobility) | Bed | Walk | Bed | Walk |
| Stairs | 1 | 3 | 1 | 5 |
| **Cognition** | | | | |
| Comprehension | 7 | 7 | 7 | 7 |
| Expression | 7 | 7 | 7 | 7 |
| Social Interaction | 6 | 6 | 7 | 7 |
| Problem Solving | 5 | 6 | 6 | 7 |
| Memory | 6 | 7 | 7 | 7 |
| **Subdomain Totals** |  |  |  |  |
| Self-Care (possible range 8-56) | 21 | 43 | 21 | 52 |
| Mobility (possible range 5-35) | 14 | 24 | 8 | 27 |
| Cognition (possible range 5-35) | 31 | 33 | 34 | 35 |
| **WeeFIM Total**  **(possible range 18-126)** | **66** | **100** | **62** | **114** |

**
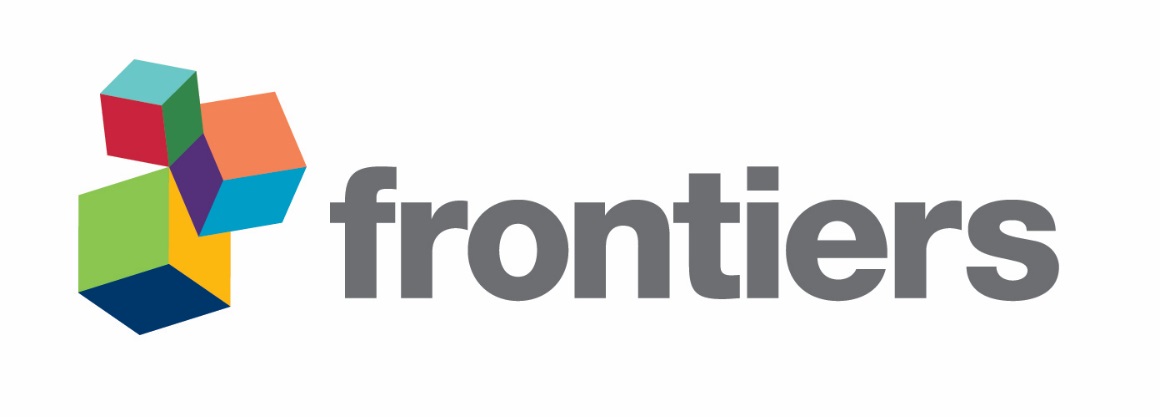
**
